# PCR-based assays for validation of single nucleotide polymorphism markers in rice and mungbean

**DOI:** 10.1186/s41065-016-0024-y

**Published:** 2017-01-26

**Authors:** Thu Giang Thi Bui, Nguyen Thi Lan Hoa, Jo-yi Yen, Roland Schafleitner

**Affiliations:** 1 Plant Resources Center, Vietnam Academy of Agricultural Sciences, An Khanh, Hoai Duc, Ha Noi, Vietnam; 2 World Vegetable Center, 60 Yi Min Liao, Shanhua, Tainan, 74151 Taiwan

**Keywords:** Single nucleotide polymorphism, Genotyping, PCR-based markers, CEL-I

## Abstract

**Background:**

Single nucleotide polymorphism (SNP) markers are the method of choice for genetic analyses including diversity and quantitative trait loci (QTL) studies. Marker validation is essential for QTL studies, but the cost and workload are considerable when large numbers of markers need to be verified. Marker systems with low development costs would be most suitable for this task.

**Results:**

We have tested allele specific polymerase chain reaction (PCR), tetra markers and a genotyping tool based on the single strand specific nuclease CEL-I to verify randomly selected SNP markers identified previously either with a SNP array or by genotyping by sequencing in rice and mungbean, respectively. The genotyping capacity of allele-specific PCR and tetra markers was affected by the sequence context surrounding the SNP; SNPs located in repeated sequences and in GC-rich stretches could not be correctly identified. In contrast, CEL-I digestion of mixed fragments produced from test and reference DNA reliably pinpointed the correct genotypes, yet scoring of the genotypes became complicated when multiple SNPs were present in the PCR fragments. A cost analysis showed that as long the sample number remains small, CEL-I genotyping is more cost-effective than tetra markers.

**Conclusions:**

CEL-I genotyping performed better in terms of genotyping accuracy and costs than tetra markers. The method is highly useful for validating SNPs in small to medium size germplasm panels.

**Electronic supplementary material:**

The online version of this article (doi:10.1186/s41065-016-0024-y) contains supplementary material, which is available to authorized users.

## Background

Single nucleotide polymorphisms (SNPs) have become the most widely used marker system for plant and animal genetic analyses. SNP arrays are available for a number of plant and animal species [1] and tools combining SNP detection and genotyping such as genotyping by sequencing (GBS) made the use of these markers in genetic studies feasible and affordable for virtually any organism, including non-model species (recently reviewed by [2]). Quantitative trait loci (QTL) analyses may yield a considerable number of markers that require verification of their correctness and diagnostic capacity to predict a phenotype. Depending on the number of markers to be tested, SNP marker validation can be even more costly and laborious than the genotyping experiment itself.

Several assay types are available for verifying SNP markers generated by GBS, SNP chips or similar technologies. Cost-effective commercial SNP assays are available [3], but the cost saving promised by these assays are only attained in routine genotyping, when large sample numbers are analyzed with the same assay. For validation, the number of genotypes used for testing a candidate marker is typically small, and only a few of these markers will be chosen for routine genotyping, while most markers will be discarded. In this situation, the high development costs for commercial assays are not compensated for by their low running costs, making these markers an expensive option for validation experiments. What is more, commercial SNP genotyping tools such as KASP or Taqman assays require specialized laboratory equipment, which might not be available in every genotyping lab, especially not in developing countries. Consequently, for marker validation, simple SNP genotyping tools with low development costs are preferred.

Conversion of SNP markers to cleaved amplified polymorphic sequence (CAPS) markers is often used as a PCR-based method to genotype SNP markers [4], but similar to commercial kits, the method might not be practical for SNP validation. First, only a fraction of SNP markers can be converted to CAPS markers, and second, the restriction enzymes for CAPS assays cause similarly high development costs like commercial assays. dCAPS markers overcome the need for the SNP to fall within a restriction enzyme recognition site and eliminate the limitation that only a fraction of SNPs can be converted to dCAPS [5], but the marker development costs remain high, as a restriction enzyme is required to distinguish the SNP alleles. PCR with allele-specific primers of different length has been proposed as a simple and cost-effective tool for PCR-based SNP genotyping [6]. This method is very cheap and easy to perform, but may be constrained by the sequence context around the SNP, which restricts the options for primer design. Similarly, tetra-primer amplification has been described as an efficient low cost method for genotyping SNP markers [7–9]. This method uses two locus-specific outer primers that asymmetrically flank the SNP under investigation, and two allele-specific inner primers, which produce a larger fragment for one allele, and a smaller fragment for the second allele. The bands of different size produced by one inner and outer primer pair can be easily detected on polyacrylamide or agarose gels [8]. To increase the specificity of the genotyping assay, an additional mismatch base near the 3′ end of the allele-specific primers can be added [8, 9]. The method may not work well for SNPs in cytosine and guanine-rich DNA regions, and restrictions for choosing the inner (allele-specific) primers may limit the assay performance and require laborious adjustments and assay optimization [10].

SNP genotyping using the single strand specific nuclease CEL-I has been proposed previously [11, 12]. The mismatch-specific nuclease CEL-I is extracted at low cost by ammonium sulfate precipitation from common celery [13, 14]. It detects mismatches in DNA double strands with high sensitivity and is commonly used to identify point mutations in methods known as TILLING and Eco-TILLING [15]. CEL-I was used to genotype SNPs present in mixtures of relatively large PCR fragments (2,000 bp) derived from two individuals after denaturation and re-naturation of the fragments [11]. Previously reported protocols require stopping the enzyme reaction by adding EDTA, or even removing excess salt and concentrating the samples through precipitation, making the method relatively laborious [11].

Rice is the most important field crop in Asia and mungbean is increasingly used as a rotation crop in rice production systems. Marker-assisted selection is routinely performed in rice breeding and becomes also popular for mungbean [16, 17]. Therefore, the present case study aimed to evaluate the performance and costs of low cost PCR-based SNP assays on these two crops. We have tested allele-specific PCR, tetra PCR and CEL-I digestion for their capacity to correctly validate the presence of SNPs previously identified in a rice germplasm panel genotyped by a GoldenGate assay, and in a bi-parental mungbean population, where SNPs were identified by GBS. Special consideration was given to streamline the assays to keep costs and labor requirements as low as possible. An ideal assay should have no or only minimal need for optimization, enabling designing and running a large number of assays for validation at minimal effort and cost. Therefore, in this study, no optimization and primer redesign was performed before assessing the performance and costs of the marker assays.

## Methods

### Plant material and SNP markers

In a previous work, a set of 26 local Vietnamese *Oryza sativa* spp. *japonica* and spp. *indica* rice cultivars held in the National Crop Genebank of the Plant Resources Center of the Vietnam Academy of Agricultural Sciences, Hanoi, Vietnam, were submitted to genetic diversity analysis at the Plant Breeding, Genetics and Biotechnology Division of the International Rice Research Institute, Los Baños, Philippines, using a 288 SNPs sub-set of the 384-SNP GoldenGate chip on a Fluidigm EP1 system according to [18]. From the genotyping results, five SNPs were chosen at random for validation with allele-specific primers, tetra markers and CEL-I genotyping (Table 1). In total 10 out of the 26 lines (R1, R4, R5, R7, R10, R15, R17, R18, R20 and R25) were chosen for SNP validation by sequencing, as these lines, according to the GoldenGate assay, displayed different combinations of the 5 SNPs in homozygote or heterozygote state.

**Table 1:** Outer and inner primer for tetra markers for rice. The outer primers were also used for producing PCR fragments for CEL-I genotyping. The fragment length refers to the fragments produced with the outer primers

Locus name	Primer name	Frag. length	Outer Fwd	Inner Fdw	Inner Rev	Outer Rev
4:22435296	Tetra_1	310	GGAACATGTCGCATTCACAGT	GCAGGTAAAACAGGTTAGG	AATTGATGCCTGACTGGCTGACT	CGACAGGGAGAAATCCTGAA
5:44806	Tetra_2	302	ACACATCGATCCAGCTTACCA	TAAGCTTCAGGCTCTAGCTTC	GTGAGAAAGTAAAGGAC	GCCTTGCAGGCTCCTATTTT
6:14504992	Tetra_3	288	CGCCAAGAAAATGTGCAGTA	TTCTCCTTGTGCCAGATAG	CACCACGGAGGTTTCTAAT	CCTTGCCCTGTTATTCCTGA
7:3650191	Tetra_4	222	AGTGCCTGCATGATTGAACA	CTGAAGTGAAAACAGAGC	GTTTATTTCAAACGGGCG	CAAGTTTGTAGGTGCTGACCA
9:6982338	Tetra_5	201	GCGGAATTACACTGTTTTTGG	TGTACTTGTATTTGCCGCTC	CCCTAAGATGTCTTAAAAATA	TTCTTGGGGTTGGCTACAAG

DNA of mungbean (*Vigna radiata*) lines V2802, NM92, NM94 and of the wild mungbean line TC1966 (*Vigna radiata* var. *sublobata*)*,* from F_7_ progenies of V2802 x NM94 and F_12_ progenies of TC1966 x NM92 was received from the World Vegetable Center mungbean breeding program. Putative SNP markers were identified in populations V2802 x NM94 and TC1966 x NM92 by genotyping by sequencing [17]. Eight putative SNPs detected in this effort were selected at random for testing tetra markers and CEL-I genotyping (Table 2). The SNPs that were verified by sequencing, tetra markers and CEL-I on the mapping parents, were validated in 139 F_7_ progenies of V2802 x NM94 and 61 F_12_ offspring of TC1966 x NM92.

**Table 2:** Outer and inner primer for tetra markers for mungbean. The outer primer were also used for producing PCR fragments for CEL-I genotyping. The fragment length refers to the fragments produced with the outer primers

Locus name	Primer name	Frag. length	Outer Fwd	Inner Fdw	Inner Rev	Outer Rev
1:26,370,595	Tetra_6	208	CCGAAGATGTGTGATTCATG	GACGATACTTGTCCAGATAT	GAAGGGATTTTGTTAGGAGT	AGCACTCAAGATGAAAGTGATC
2:23,741,639	Tetra_7	260	ACTATCTGACCGAAAGGAA	TTGGTACCAAATTCTGCACT	ATCTTACGGTGAAGGACATT	ATCTGCTGACAGGAGAATTCA
3:11,561,441	Tetra_8	236	CCCCTAAAGCTGGAATATA	TCTTCAAAGAGCTCCTTTTG	GATTGTCACTTTGTCAAAG	TGATCATCAAGCTACACTTC
3:10,830,938	Tetra_9	275	AGCGAGGACAAGGAAGAATT	TCTGCGGTGTTCTTAGAGT	TTCCTCCAAATGCTGCAGAC	GGCAAATTATTAGGATCCTGC
5:9,090,455	Tetra_10	250	TCGCTTAACCAAGAATCGC	CCAATGCGAATCAGATG	AAAACGCACATCTGGTTCGTGT	CGACATGGTGGCAATTTGAT
7:13,713,780	Tetra_11	253	TAGCTGGTCCGTGTACTTTA	TTTCCATTGTGGGTCGTGGAGT	GCAGCAACATGTGCAACAT	GGAACTATGCTTTGGGACTT
10:3,159,416	Tetra_12	269	ATACTGGAGGGTTGTTTCTA	TAAGCGTGCGCAGCCATAAACAAG	GGGTTTTTTCGGAAATTCAAAG	CGGTCTCAGAATCATAGTCTTG
12:9,262,432	Tetra_13	215	AACCCCTTTATATAGGGTCTG	TCATCTTCTCCGGGCAGCTCCTTC	GAGGTGAAGGCACGTGACCA	TCATTCGAGGAAAGGAGAGA

DNA extraction for sequencing, analysis by allele-specific primers, tetra primers and CEL-I was done from young leaves using the CTAB protocol described by [19]. PCR-fragments for sequencing were produced from DNA of the rice and mungbean genotypes using the outer primers listed in Tables 1 and 2. The PCR fragments were submitted to Sanger sequencing at Genomics Ltd, Taiwan and the forward and reverse sequence reads were assembled and analyzed in DNAStar version 7.1.

### Allele-specific PCR

The allele-specific PCR technique was applied as described by [6]. This method uses three primers: a common forward primer and two allele-specific reverse primers, where the 3′ prime end is specific for a SNP allele. In addition, each of the allele-specific primers contained a mismatch base near the 3′ end to destabilize the hybridization with the target sequence and increase the specifity of the allele-specific primer. One allele-specific primer contained a five base random extension at the 5′ site, and the primer for the second allele contained a 15 base extension, where the five 5′ bases corresponded to the extension of the first primer. The different length of the primers should result in size differences of the amplification products derived from the different alleles visible after gel electrophoresis of the PCR fragments. Primers were designed after retrieving the SNP sequence context in the *O. sativa* reference at http://rice.plantbiology.msu.edu/cgi-bin/gbrowse/rice/ using Primer3 http://bioinfo.ut.ee/primer3-0.4.0/. The primer sequences and the amplicon sizes are shown in Table 3. PCR was performed on 20 ng genomic DNA in 15 μl reactions containing 0.2 μM of each primer, 200 μM of deoxyribonucleotides, 50 mM KCl, 10 mM Tris HCl (pH 8.3), 1.5 mM MgCl_2_ and 0.5 units of Taq DNA polymerase on a DNA Gradient PCR machine (BIORAD) with an amplification profile of initial denaturation at 95 °C for 10 min, followed by 35 cycles with 95 °C for 30 s, annealing at an assay-specific temperature from 48 °C to 65 °C for 45 s, elongation at 72 °C for 45 s and terminal elongation at 72 °C for 5 min. PCR products (2 μL/sample) were analyzed on 6% polyacrylamide gel and visualized after ethidium bromide staining under UV light.

**Table 3:** Allele-specific primers for rice

Locus name	Primer name	Fragment length	Common primer	Allele-specific primer 1	Allele-specific primer 2
4:22435296	AS1	219	CGACAGGGAGAAATCCTGAA	5’-GACTTGGCAGGTAAAACAGGTTACG-3'	5’-CACTACAAAGGCTGTGGCAGGTAAAACAGGATAGA-3'
5:44806	AS2	189	GCCTTGCAGGCTCCTATTTT	5’-AGTCAAAGCTTCAGGCTCTAGCTGC-3’	5’-CGACTAACTGTGACTAAGCTTCAGGCTCTAGCATA-3’
6:14504992	AS3	209	CCTTGCCCTGTTATTCCTGA	5’-CCAGTTTTCTCCTTGTGCCAGATTG-3’	5’-GATGTCTACTCCAGTTTTCTCCTTGTGCCAGAGAA-3’
7:3650191	AS4	293	CAAGTTTGTAGGTGCTGACCA	5’-GATCCTAACTGAAGTGAAAACAGCA3’	5’-TGCGTCATAGGATCCTAACTGAAGTGAAAACACAC-3’
9:6982338	AS5	162	TTCTTGGGGTTGGCTACAAG	5’-ACTGCTGTACTTGTATTTGCCGCAC-3’	5’-CATTGTATTTTTACGTGTACTTGTATTTGCCGATT-3

The allele-specific base, as well as the additionally introduced variant base near the 3’end are underlined. The primer extension sequences are printed in small letters. The fragment length excludes the extensions of the allele-specific primers

### Tetra marker

Outer and inner primers for tetra markers were designed in Primer3 (http://bioinfo.ut.ee/primer3-0.4.0/) using the rice and mungbean reference sequences (http://rice.plantbiology.msu.edu/cgi-bin/gbrowse/rice/ and http://plantgenomics.snu.ac.kr/mediawiki-1.21.3/index.php/Main_Page). The additional mismatch base usually introduced for tetra ARMS PCR [8] was not applied in our experiment. The outer and inner primers of the tetra markers are listed in Tables 1 and 2. The PCR reactions were set up as described for allele-specific primers. The amplification profile was 94 °C for 5 min, followed by 30 cycles of 94 °C for 30 s, 58 °C for 45 s, 72 °C for 45 s, and final extension for 7 min at 72 °C. PCR products (3 μl) were size-fractionated on 6% non-denaturing polyacrylamide gels in 0.5 × TBE buffer. After electrophoresis, the gels were stained with 5 μg/mL^−1^ ethidium bromide and the bands were visualized under ultraviolet light. Tetra markers were tested on five SNPs of rice landraces and on eight SNPs of mungbean mapping parents.

### CEL-I SNP genotyping method

**Fig. 1:**
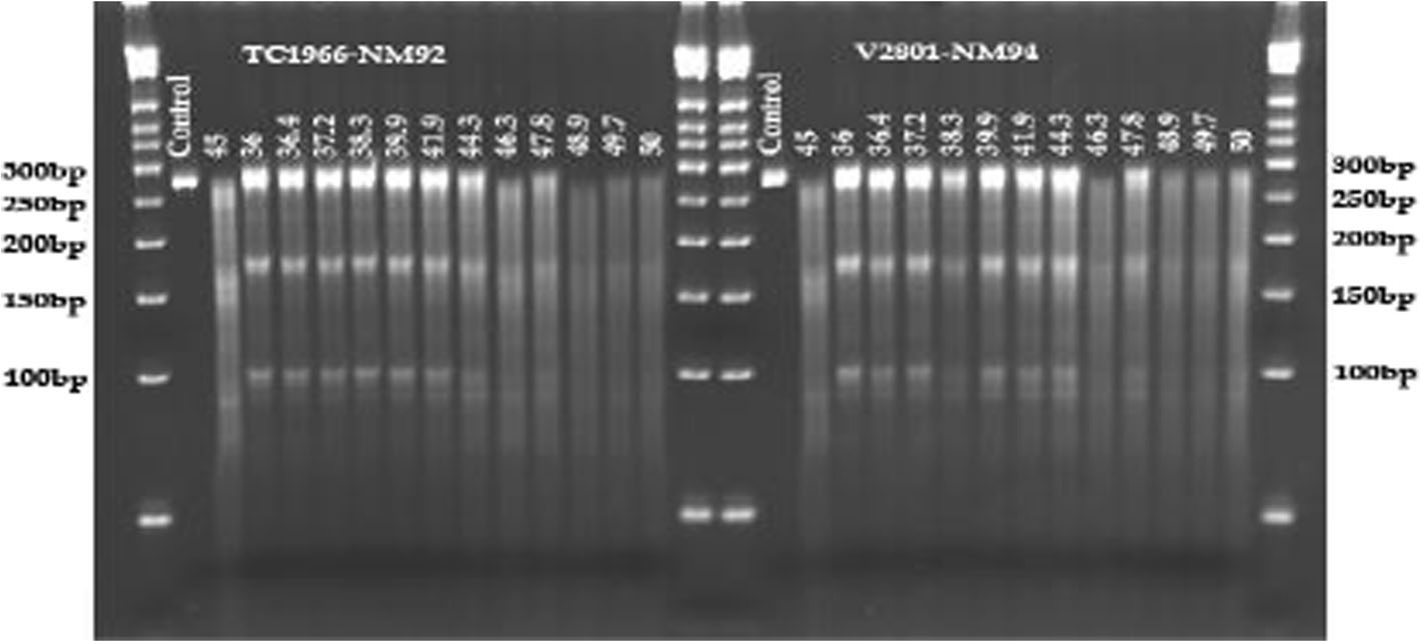
Optimization of the incubation temperature for CEL-I treatment. The SNP tested was tetra_12. Incubation at 36 ° was found to be optimal to specifically cut at the mismatch site in position 171 bp of the 269 bp fragment, producing bands with 170 and 99 bp. The additional smaller band below around 90 bp is probably due to the sequence context around the SNP (please refer to the discussion)

500 g celery was purchased at a local farmer’s market. Leaves were removed and the stems were blended in a juicer. CEL-I was enriched through (NH_4_)_2_SO_4_ precipitation as described by [15]. Desalted CEL-I extract was stored in aliquots at −80 °C. Test digestions using 0.5 to 2.5 μl CEL-I extract showed that 1 μl were optimal for genotyping. The CEL-I working solution for each reaction contained 1 μL CEL-I enzyme, 1.5 μL CEL-I buffer and 7.5 μL distilled deionized water. 10× CEL-I buffer contained 1 M MgSO_4_, 1 M HEPES pH 7.5, 2 M KCl, 10% Triton X-100, and 20 mg/ml BSA. PCR fragments produced with the outer primers of the tetra markers described above were used for CEL-I genotyping. PCR products derived from different plant lines were mixed in a ratio of a 1:1 ratio (v/v). For genotyping progenies of mapping parents, two assays per sample were prepared. In one assay the DNA of the progeny was mixed with DNA of mapping parent one, and in the second assay it was mixed with DNA of mapping parent two. For genotyping mapping parents, also two assays per locus were performed. In one assay the DNA of one mapping parent was mixed with the DNA of the other parent, and in the second assay the DNA of each mapping parent was genotyped separately. When the SNP base was identical with the base present in the DNA mixed with the sample, no cut by CEL-I was obtained, while in the presence of one or more polymorphic bases, CEL-I could introduce a cut into the fragment. This way, the reference, variant or heterozygote genotype at SNP base located in the tested PCR fragments could be determined. For the assay, the DNA double strands were denaturated by incubation at 99 °C for 10 min, reannealing the stands by cooling down to 70 °C, and subsequently reducing the incubation temperature from 70 °C to 49 °C in 70 cycles with −0.3 °C intervals per 20 s. 10 μl ice-cold CEL-I working solution was added to the sample on ice, mixed and incubated at different temperatures. For PCR fragments of less than 300 bp, the usually suggested CEL-I treatment incubation temperature of 45 °C was too high and yielded strong background and weak bands, while a temperature of 36 °C seemed to be suitable to yield bands after specifically cutting mismatch bases (Fig. 1). For the CEL-I genotyping in the current study, the samples were incubated at 34 °C for 10 min and then cooled down to −20 °C. Electrophoresis and DNA fragment visualization was performed as described for tetra markers. A simplified version of CEL-I digestion was performed by performing PCR on DNA mixtures, rather than mixing fragments post-PCR.

## Results

### SNP validation in rice by sequencing and testing allele-specific PCR, tetra markers and CEL-I genotyping

In total, five SNPs detected through the 384-SNP GoldenGate (*Indica* x *Indica*) array were selected for verification. Before testing PCR-based genotyping on these SNPs, the SNP data obtained from the GoldenGate array were verified by sequencing on 11 rice landraces (Table 4).

**Table 4:** SNPs among rice landraces selected from Golden Gate genotyping data

Golden Gate data				Sequencing data										
Locus name	Primer name	Ref	Var	R1	R4	R5	R7	R10	R15	R17	R18	R20	R21	R25
4:22435296	tetra_1	G	A	G	G	G	G	G	A	G	G	G	G	G
5:44806	tetra_2	C	G	G	C	C	S	C	C	C	C	C	C	C
6:14504992	tetra_3	G	A	G	G	G	G	G	G	G	G	G	R	A
7:3650191	tetra_4	A	C	C	A	A	A	A	A	A	A	A	A	A
9:6982338	tetra_5	C	T	C	C	C	C	C	C	C	C	C	C	C

Ref.: reference sequence, Var.: variant. R1 – R25: rice landrace accession number

**Fig. 2:**
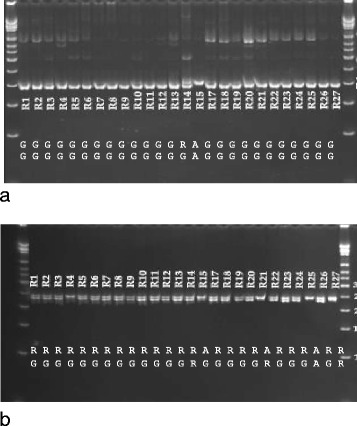
Genotyping of rice using allele-specific markers. **a**) Tetra_1, **b**) Tetra_3. The SNP bases are indicated at the bottom of the gel images. The top row shows the allele-specific PCR results, and the SNP array genotyping or sequencing results are shown in the bottom row

Allele-specific PCR, in addition to the allele-specific bands, yielded several secondary bands for tetra_1, but still genotypes were predicted correctly except for one case (R14). The allele-specific markers for tetra _3 predicted heterozygote genotypes for 21 out of 26 genotypes (Fig. 2) and revealed the correct genotype only in one sample (R25). The other allele-specific primers did not produce bands that distinguished the SNP alleles (data not shown). This result showed that allele-specific primers as designed for this experiment are not useful for genotyping SNPs in rice samples. Allele-specific primers were not tested on mungbean.

**Fig. 3:**
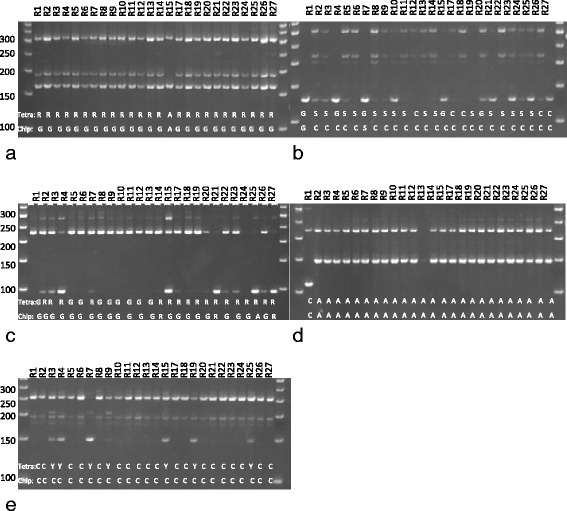
Genotyping selected rice accessions by tetra markers: **a**) tetra_1, **b**) tetra_2 and **c**) tetra_3, **d**) tetra_4, **e**) terta_5. The SNP bases indicated by the allele specific PCR detected by SNP array genotyping or sequencing are indicated on the gel pictures

**Fig. 4:**
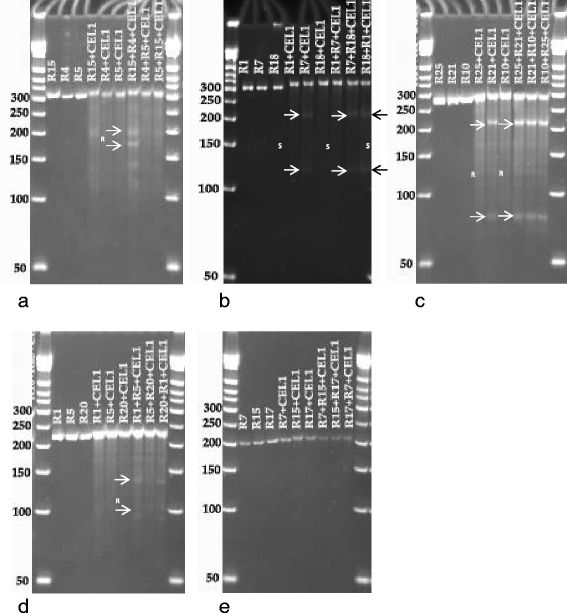
CEL-I genotyping of rice accessions with tetra_1 (**a**), tetra_2 (**b**), tetra_3 (**c**),tetra_4 (**d**), and tetra_5 (**e**). The fragments produced by CEL-I by cutting at heterozygous loci (b for R7, c for R21) and at SNP loci are indicated with arrows and listed in Table 4

Testing tetra markers on these SNPs showed that markers tetra_1, 2 and 3 had the tendency to show heterozygote genotypes, although the accessions were homozygote at the tested loci. The A variant for the G/A SNP in tetra_1 was correctly recognized by tetra markers, while most G variants were erroneously genotyped as G/A heterozygotes (Fig. 3). The G/A SNP of tetra_1 was located between G residues, which might have affected the specific initiation of amplification by primer for the G-allele. The SNP in tetra_2 was located in a repeat sequence (AGCTTG), and the C-specific primer initiated polymerase chain reaction at the target site and at the second AGCTTG motif located 13 bp upstream the target sequence in all samples with the C allele present. In addition, the inner primer for the G allele was not specific and amplified a fragment in all samples suggesting erroneous heterozygote genotypes. Tetra marker tetra_3 gave in many cases erroneous heterozygote genotypes, as the inner reverse primer specific for the A allele yielded products, albeit at low amounts, when only the G allele was present. Only tetra markers tetra_4 gave the correct genotypes and detected the C-variant in R1. Tetra_5 scored seven homozygous samples. In contrast, CEL-I genotyping reliably detected all heterozygote and homozygote SNPs correctly (Fig. 4). The G/A SNP between R4 and R15, as well as the heterozygote G/C in R7 and the G/C SNP between R18 and R1 were correctly detected. The same with tetra_3, the heterozygote base in R21 and the SNP among lines R10 and R25 were detected. Also the SNPs in tetra 4 between R1 and R25 and R1 and R20 were correctly detected. Tetra-5 did not contain any SNP, therefore no CEL-I cut was observed in this fragment.

### SNP validation in mungbean by sequencing and testing tetra markers and CEL-I genotyping

In total 8 putative SNPs obtained from GBS (Table 5) were chosen for validation through sequencing, and testing of tetra-markers and CEL-I analysis. Sequencing confirmed the SNPs for tetra_6, 7, 9, 12 and 13, but not for tetra_8, 10 and 11 (Table 5). In addition to the SNPs previously found by genotyping by sequencing, Sanger sequencing of the PCR fragments produced with the outer tetra primers resulted in additional SNPs for tetra 6, 7, 9, 10 and 13. The outer primers for tetra_11 did not yield any product from TC1966 and therefore could not be sequenced. Tetra-11 was monomorphic between NM94 and V2802 and tetra_8 was monomorphic between all mapping parents. The amplicon of tetra_10 contained several heterozygous sites in all mapping parents, but sequencing did not corroborate the presence of the G/A polymorphism predicted by GBS. This sequencing exercise showed that 3 out of the 8 selected SNPs predicted by GBS were erroneous, probably due to wrong mapping of GBS tags to the reference sequence, and due to insufficient sequencing depth.

**Table 5:** Mungbean SNPs detected by genotyping by sequencing among the mapping parents NM92, NM94, TC1966 and V2802 and targeted for genotyping by tetra markers and CEL-I

GBS data							Sequencing data			
Locus name	Primer name	SNP Position in PCR fragment	NM92	TC1966	NM94	V2802	NM92	TC1966	NM94	V2802
1:26,370,595	tetra_6	144	T	A	T	T	T	A	T	T
2:23,741,639	tetra_7	181	T	T	T	A	T	T	T	A
3:11,561,441	tetra_8	-	C	G	C	C	C	**C**	c	c
3:10,830,938	tetra_9	200	G	G	G	A	G	G	G	A
5:9,090,455	tetra_10	-	G	A	G	G	G,	**G**	G	G
7:13,713,780	tetra_11	-	A	A	A	G	A	A	A	**A**
10:3,159,416	tetra_12	171	C	G	C	G	C	G	C	G
12:9,262,432	tetra_13	140	T	C	C	C	T	C	C	C

The numbers refer to the position of the SNP in the PCR fragment obtained from the mapping parents using the outer tetra primers. SNPs that could not be validated by sequencing are labeled in bold

**Fig. 5:**
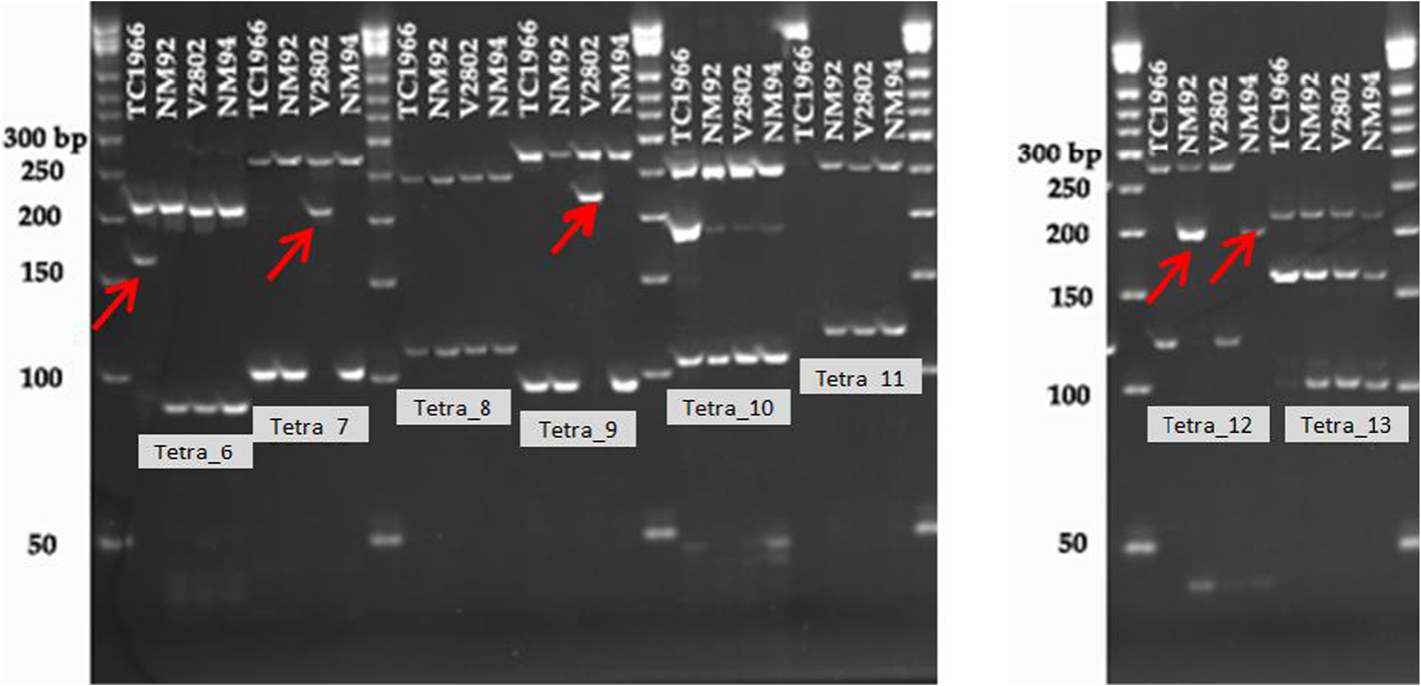
Test of tetra markers tetra-6, 7, 8, 9, 10 11, 12 and 13 on 4 mungbean mapping parents TV1966, NM92, V2802 and NM94. The bands indicating the SNP base are labeled with arrows

All SNPs, including those that could not be confirmed by Sanger sequencing were submitted to testing by tetra marker and CEL-I genotyping. Tetra markers applied without an additional mismatch base genotyped four out of the five sequence-corroborated SNPs correctly. Only for tetra_13 did the tetra marker fail to reveal the correct genotype (Fig. 5). Genotyping of F7 populations TC1966 x NM92 and/or V2802 x NM94 with tetra_6, 7, 9 and 12 showed the segregation of the SNP genotype in the populations (Additional file 1) and suggested that the tetra markers were appropriate for genotyping populations.

**Fig. 6:**
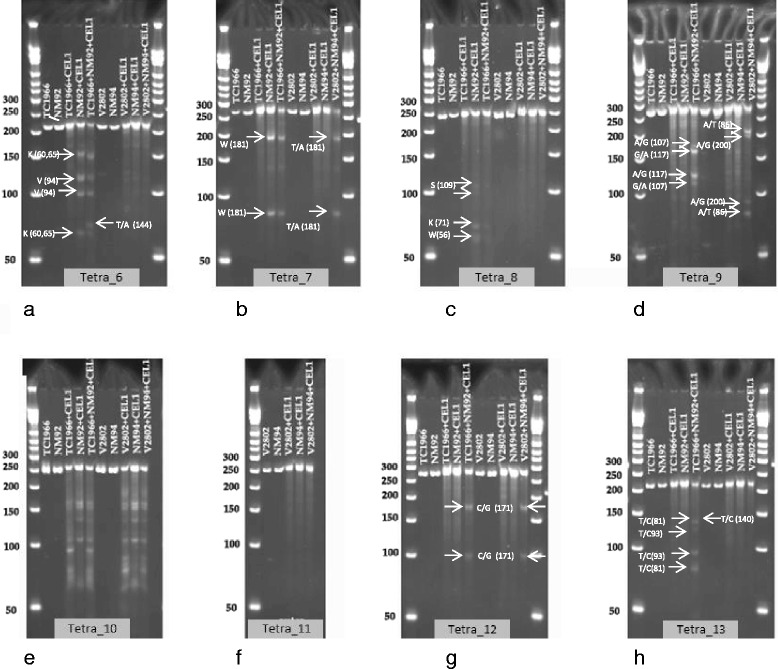
CEL-I test genotyping on pure and mixed DNA of 4 mungbean mapping parents for tetra_6 (**a**), tetra_7 (**b**), tetra_8 (**c**), tetra_9 (**d**), tetra_10 (**e**), tetra_11 (**f**), tetra_12 (**g**), and tetra_13 (**h**). CEL-I cuts at mismatch sites. Mismatches can appear at heterozygote sites in one genotype (as in NM92 with tetra_6 (**a**) in positions 60/65 and 94, or at SNP sites when PCR fragments from homozygote mapping parents are mixed, as in tetra_6 at position 144 of TC1966/NM92, or in tetra_12 (**g**). The SNPs among the mapping parents and the heterozygote loci causing the CEL-I fragments are labeled on the figures and listed in Tables 5 and 6

The outer primers of the tetra markers were used to amplify fragments from genomic DNA of the mapping parents containing the putative SNP sites. PCR amplification products from TC1966 were pooled with those from NM92, and products from NM94 were pooled with those from V2802. The pure samples and the mixtures were submitted to CEL-I treatment. In un-pooled PCR fragments obtained from the four mapping parents, CEL-I cutting was observed at the heterozygous bases in fragments of tetra_6, 7 and 8 in NM92 and tetra_10 in all mapping parents (Fig. 6, Table 6). High background was obtained with tetra_9 from all mapping parents. This fragment contained microsatellite-like motifs, which could have led to destabilization of the DNA double strand of the short fragments and could have transiently created cutting sites for CEL-I. Further reduction of the reaction temperature to avoid this effect was not tried. CEL-I treatment of mixtures of SNP-containing PCR fragments of the mapping parents resulted in the expected DNA fragments for all loci (Fig. 6, Tables 5 and 6). In addition to the cuts at heterozygote sites, in mixtures of tetra_6 PCR fragments from NM92 and TC1966, an additional cut was produced at position 144 (Fig. 6a). In tetra_7, as expected from the sequencing data, the heterozygous site at position 181 of the PCR fragment from NM92 caused a CEL-I cut, resulting in a 181 and a 79 bp band, while in mixtures of NM94/V2802 PCR fragments CEL-I genotyping correctly showed the T/A polymorphism in position 181 of the fragment (Fig. 6b, Tables 5 and 6). The monomorphic locus tetra_8 remained un-cut by CEL-I, cuts were introduced at the heterozygous sites in NM92 (Fig. 6c, Table 6). Tetra_10 gave a complex pattern due to the presence of multiple SNPs in the PCR fragment (Table 6), while CEL-I restriction of tetra_11 fragments gave no cuts, as expected for these monomorphic fragments. Mixtures of fragments of tetra_12 from TC1966 and NM92, as well as from V2802 and NM94 gave the expected cuts at the SNP position 171 of the PCR fragment. Similarly mixtures of PCR fragments of tetra_13 from TC1966 and NM92 gave the expected pattern of cut bands at the SNP site.

**Table 6:** Mungbean SNPs found by Sanger sequencing of PCR fragments produced with the outer tetra marker primers. These SNPs are located nearby the SNPs that were detected by genotyping by sequencing

Locus name	Primer name	NM92	TC1966	NM94	V2802
1:26,370,595	tetra_6	60:K, 65:K, 94: V, 95:G	64:T, 95:A	none	none
2:23,741,639	tetra_7	44:A, 127:T, 181: W	44:T, 127: C	127: C	127: C,
3:11,561,441	tetra_8	56: M, 71:Y, 109:W	none	none	none
3:10,830,938	tetra_9	107:A, 117:G	107:G, 117:A	86:A	86:T
5:9,090,455	tetra_10	71:T, 77:T, 95:A, 118:G, 138:C, 213:Y,	71:C, 77:Y, 95:S, 118:K, 138:Y, 213:C,	71:Y, 77:Y, 95:A, 102:W, 149:S, 178:S, 213:Y, 225:C	24:C, 71:Y, 77:Y, 168:W, 178:S
7:13,713,780	tetra_11	none	none	none	none
10:3,159,416	tetra_12	none	none	none	none
12:9,262,432	tetra_13	81:C, 93:T	81:T, 93:C	none	none

CEL-I genotyping on populations was tested for tetra_7, 9 and 12 on F_7_ families of V2802 x NM94 and for tetra_12 on F_12_ families of populations TC1966 x NM92 (Additional file 2). Instead of mixing the PCR fragments of the families with the fragments of the mapping parents, the PCR mastermix was spiked with either parent 1 or parent 2 DNA before amplification. For each sample, two amplification reactions were performed, one with parent 1 and another with parent 2-spiked DNA in order to detect heterozygous SNPs. Scoring of CEL-I genotyping was simple on amplicons with a single SNP like in tetra_12, but was also feasible when additional SNPs or heterozygous sites were present, like in tetra_9. By comparing the genotypes of the parent 1- and parent 2-spiked families, homozygote and heterozygote genotypes could be distinguished easily (Additional file 2). For example, for tetra_9 samples 14 and 128 showed the cut bands in both, NM94 and V2802-spiked CEL_I reactions, indicating that the samples are heterozygote for the tetra_9 SNP (Additional file 2).

### Validation of SNP markers - Cost and working time considerations

Using commercial SNP assays for marker validation is relatively expensive. For mapping experiments dealing with complex traits involving many loci and numerous markers associated with them, the validation costs for candidate markers may surpass the genotyping costs. Table 7 shows a comparison of reagent costs and working time for different marker systems including CAPS (dCAPS), tetra markers, and CEL-I assays. Allele-specific markers were excluded from the calculation, as they did not perform sufficiently well in our experiments to be considered as a validation tool. DNA extraction costs were not considered, as these costs were equal for all listed assays. Many different restriction enzymes are required to cover SNPs in different sequence contexts, therefore developing new CAPS or dCAPS markers often requires purchasing new restriction enzymes. For the calculation in Table 7 we assumed that for every third SNP a new kind of restriction enzyme has to be purchased. The cost of supplies to develop a marker assay are clearly highest for CAPS markers, followed by tetra markers and CEL-I genotyping. The time for designing and testing the markers is assumed to be higher for tetra and CAPS markers than for CEL-I genotyping. Mainly due to the restriction enzyme costs, CAPS development remains the most expensive system. For routine genotyping, CEL-I would be the most expensive and laborious option, as two assays must be performed for each locus to distinguish homozygote from heterozygote variants. Therefore, for marker validation on a small number of samples, where mostly marker development costs play a role, CEL-I genotyping seems to be cheapest; for genotyping a larger sample, tetra markers are more cost-effective.

**Table 7:** Estimation of development and running costs for (d)CAPS, tetra marker and CEL-I-based genotyping (in US$)

Development costs			
	CAPS (dCAPS)	Tetra marker	CEL-I
Primer	7.5	15	7.5
Restriction enzyme	30		1
PCR reagents	0.48	0.48	0.96
Gel electrophoresis and DNA band detection	0.06	0.06	0.12
TOTAL	38.04	15.54	9.58
Work load			
Approximate working time for development 1 assay (h)	0.25	0.25	0.1
Working time for testing	0.12	0.08	0.12
TOTAL	0.37	0.33	0.22
Routine analysis			
Hands-on time per assay (assuming 96 assays per run, in h)	0.03	0.025	0.06
Reagent costs per assay	0.13	0.105	0.19

## Discussion

Validation of molecular markers for breeding may require testing many candidate markers. Only a few of them will be adopted for marker-assisted selection, while most markers may be discarded. Consequently, marker validation may constitute a major cost factor. In order to keep validation costs low and to avoid the need for expensive instruments, SNPs are often converted to PCR-based markers. We have tested allele-specific PCR, tetra markers and a CEL-I based genotyping method for validating selected SNP markers derived from an array-based SNP genotyping study on rice and from a GBS experiment in mungbean. In parallel, the targeted SNPs were validated by sequencing. As expected, not all SNPs pinpointed by the GBS experiment were true SNPs. Two of the putative SNPs of mungbean were in fact monomorphic. In mungbean, chromosomal rearrangements or an indel in the GBS tag in comparison to the reference sequence [20] led to erroneous mapping and resulted in false positive SNPs. In general array-based genotyping is considered more accurate than GBS, nevertheless GBS is widely used for genotyping rice [21], mungbean [17] and other crops. Shallow sequencing and erroneous mapping of sequence tags in GBS can yield wrong genotypes, but GBS is more cost effective than array-based genotyping and has the additional advantage that no previous information on the SNPs present in the samples is required [22].

Allele-specific PCR was unsuitable to genotype SNPs in the rice samples without assay optimization and therefore was abandoned. The accuracy of tetra marker genotyping was affected by the sequence context. Introduction of a sequence mismatch near the 3′ end of the primers to increase stability might have overcome this problem [8] but was not tested, as the study addressed only the least work- intensive SNP genotyping. Optimization of the SNP assays beyond gradient PCR to determine the optimal annealing temperature for the allele-specific and tetra primers was avoided, as the study aimed to assess the performance of the assays without laborious modifications.

Previously published CEL-I protocols proved to be useful for SNP validation and genotyping [11, 12], but could be simplified to make the assays cheaper and easier to use. Stopping the CEL-I reaction by adding EDTA, as described by published protocols, was found to be unnecessary; similarly, the previously suggested desalting of the reaction mixtures prior to loading the DNA on the gel was eliminated, without affecting the scoring of the bands. Lowering the reaction temperature of CEL-I below 36 °C was essential to reduce the background. CEL-I genotyping for validation of SNP markers was the least costly method to develop, but the method required more resources per locus than tetra markers for genotyping large numbers of samples. Either PCR fragments containing the SNP have to be mixed after PCR, or DNA to be genotyped must be spiked with reference DNA. For each sample and locus, two CEL-I reactions have to be performed to distinguish homozygote from heterozygote genotypes, making the genotyping more expensive and more laborious. From our perspective, mixing DNA before PCR is the preferred CEL-I genotyping method, as spiking of the PCR master mix is less laborious and safer than handling the PCR fragments for combination after amplification. CEL-I genotyping also was more reliable than tetra markers, which may be affected by the sequence context of the SNP and require additional optimization of the assay, leading to longer development times and higher costs. However, CEL-I based genotyping was tedious on heterozygote samples, as the banding pattern became quite complex and difficult to score with increasing heterozygosity of the mapping parents.

It can be expected that more modern genotyping tools such as KASP markers [23] are more accurate than the methods presented here, but require equipment such as a plate reader that might not be available to all users of molecular markers. By adopting an improved protocol for CEL-I genotyping we wanted to provide a simple and cheap method for genotyping for laboratories that lack specialized equipment.

## Conclusions

Allele-specific PCR was not suitable to genotype SNPs in rice. The genotyping accuracy of tetra markers was affected by low allele specifity of the inner primers, repeat sequences and nucleotide runs. In contrast, genotyping by using CEL-I digestion at low incubation temperatures performed well and was independent of the sequence context around the SNP. It was also more cost effective than tetra markers, as long as the number of tested samples remained small. Additional SNPs to the SNP selected for genotyping present in the PCR fragment can make scoring of CEL-I genotypes tedious. Therefore, PCR fragments for CEL-I genotyping should be kept small to minimize the presence of additional SNPs.

## Additional files

Additional file 1: Genotyping segregating mungbean populations with tetra markers. (PDF 219 kb)
Additional file 2: CEL-I genotyping in segregating mungbean populations TC1966 x NM92 and V2802 x NM94. (DOCX 23250 kb)

## Abbreviations

ARMS PCR: Amplification-refractory mutation system PCR
BSA: Bovine serum albumin
CAPS: Cleaved amplified polymorphic sequence
CTAB: Cetyl trimethylammonium bromide
dCAPS: Derived cleaved amplified polymorphic sequences
EDTA: Ethylenediaminetetraacetic acid
GBS: Genotyping by sequencing
HEPES: 4-(2-hydroxyethyl)-1-piperazineethanesulfonic acid
KASP: Kompetitive Allele Specific PCR
PCR: Polymerase chain reaction
QTL: Quantitative trait locus
SNP: Single nucleotide polymorphism

## References

[1] Ganal MW, Polley A, Graner EM, Plieske J, Wieseke R, Luerssen H, Durstewitz G (2012). Large SNP arrays for genotyping in crop plants. J Bioscience.

[2] Andrews KR, Good JM, Miller MR, Luikart G, Hohenlohe PA (2016). Harnessing the power of RADseq for ecological and evolutionary genomics. Nat Rev Genet.

[3] Semagn K, Babu R, Hearne S, Olsen M (2014). Single nucleotide polymorphism genotyping using Kompetitive Allele Specific PCR (KASP): overview of the technology and its application in crop improvement. Mol Breeding.

[4] Thiel T, Kota R, Grosse I, Stein N, Graner A (2004). SNP2CAPS: a SNP and INDEL analysis tool for CAPS marker development. Nucleic Acids Res.

[5] Neff MM, Neff JD, Chory J, Pepper AE (1998). dCAPS, a simple technique for the genetic analysis of single nucleotide polymorphisms: experimental applications in Arabidopsis thaliana genetics. Plant J.

[6] Gaudet M, Fara A-G, Beritognolo I, Sabatti M. Allele-specific PCR in SNP genotyping. In: Komar AA, editor. Single Nucleotide Polymorphisms: Methods and Protocols. New York: Humana Press; 2009. p. 415–24. 10.1007/978-1-60327-411-1_26 19768609

[7] Ye S, Humphries S, Green F (1992). Allele specific amplification by tetra-primer PCR. Nucleic Acids Res.

[8] Ye S, Dhillon S, Ke X, Collins AR, Day IN (2001). An efficient procedure for genotyping single nucleotide polymorphisms. Nucleic Acids Res.

[9] Chiapparino E, Lee D, Donini P (2004). Genotyping single nucleotide polymorphisms in barley by tetra-primer ARMS-PCR. Genome.

[10] Medrano RFV, de Oliveira CA (2014). Guidelines for the tetra-primer ARMS–PCR technique development. Mol Biotechnol.

[11] Raghavan C, Naredo MEB, Wang H, Atienza G, Liu B, Qiu F, McNally KL, Leung H (2007). Rapid method for detecting SNPs on agarose gels and its application in candidate gene mapping. Mol Breeding.

[12] Galeano CH, Gomez M, Rodriguez LM, Blair MW (2009). CEL-I nuclease digestion for SNP discovery and marker development in common bean (L.). Crop Sci.

[13] Oleykowski CA, Mullins CRB, Godwin AK, Yeung AT (1998). Mutation detection using a novel plant endonuclease. Nucleic Acids Res.

[14] Yang B, Wen X, Kodali NS, Oleykowski CA, Miller CG, Kulinski J, Besack D, Yeung JA, Kowalski D, Yeung AT (2000). Purification, cloning, and characterization of the CEL-I nuclease. Biochemistry.

[15] Till BJ, Burtner C, Comai L, Henikoff S (2004). Mismatch cleavage by single-strand specific nucleases. Nucleic Acids Res.

[16] Duitama J, Silva A, Sanabria Y, Cruz DF, Quintero C, Ballen C, Lorieux M, Scheffler B, Farmer A, Torres E, Oard J (2015). Whole genome sequencing of elite rice cultivars as a comprehensive information resource for marker assisted selection. PLoS One.

[17] Schafleitner R, Huang SM, Chu SH, Yen JY, Lin CY, Yan MR, Krishnan B, Liu MS, Lo HF, Chen CY, Chen LFO, Wu DC, Bui TGT, Ramasamy S, Tung C-W, Ramakrishnan N (2016). dentification of single nucleotide polymorphic markers associated with resistance to bruchids (*Callosobruchus* spp.) in wild mungbean (*Vigna radiata* var. *sublobata*) and cultivated *V. radiata* through genotyping by sequencing and quantitative trait locus analysis. BMC Plant Biol.

[18] Thomson MJ, Zhao K, Wright M, McNally KL, Rey J, Tung C-W, Reynolds A, Scheffler B, Eizenga G, McClung A (2012). High-throughput single nucleotide polymorphism genotyping for breeding applications in rice using the BeadXpress platform. Mol Breed.

[19] Doyle JJ (1987). A rapid DNA isolation procedure for small quantities of fresh leaf tissue. Phytochem Bull.

[20] Kang YJ, Kim SK, Kim MY, Lestari P, Kim KH, Ha B-K, Jun TH, Hwang WJ, Lee T, Lee J, Shim S, Yoon MY, Jang YE, Han KS, Taeprayoon P, Yoon N, Somta P, Tanya P, Kim KS, Gwag J-G, Moon J-K, Lee J-H, Park B, Bombarely A, Doyle JJ, Jackson SA, Schafleitner R, Srinives P, Varshney RK, Lee S-H (2014). Genome sequence of mungbean and insights into evolution within *Vigna* species. Nature Comm.

[21] Tang W, Wu T, Ye J, Sun J, Jiang Y, Yu J, Tang J, Chen G, Wang C, Wan J (2016). SNP-based analysis of genetic diversity reveals important alleles associated with seed size in rice. BMC Plant Biol.

[22] Elshire RJ, Glaubitz JC, Sun Q, Poland JA, Kawamoto K, Buckler ES, Mitchell SE (2011). A robust, simple genotyping-by-sequencing (GBS) approach for high diversity species. PLoS One.

[23] Liu Z, Zhu C, Jiang Y, Tian Y, Yu J, An H, Tang W, Sun J, Tang J, Chen G, Zhai H (2016). Association mapping and genetic dissection of nitrogen use efficiency-related traits in rice (*Oryza sativa* L.). Funct Integr Genomics.

