# Supplementary material for: PCR-based assays for validation of single nucleotide polymorphism markers in rice and mungbean
Source: Hereditas. 2017 Jan 26;154:3. doi: 10.1186/s41065-016-0024-y (PMC5270362; doi:10.1186/s41065-016-0024-y)
Supplement: Additional file 1: — Genotyping segregating mungbean populations with tetra markers. (PDF 219 kb) [file 41065_2016_24_MOESM1_ESM.pdf]

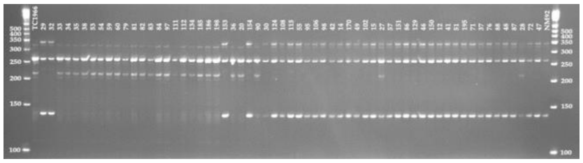

a)

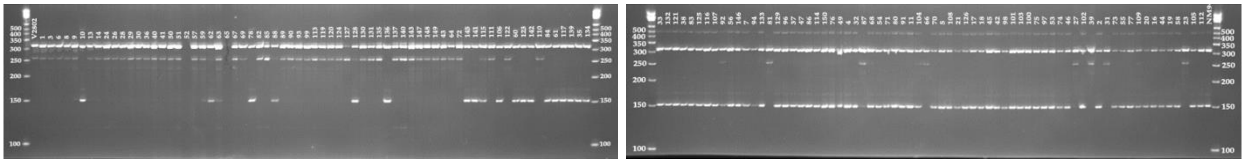

b)

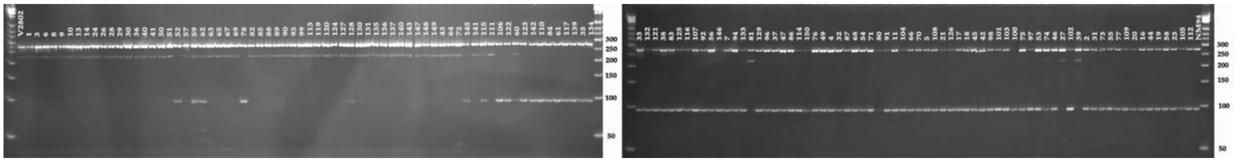

c)

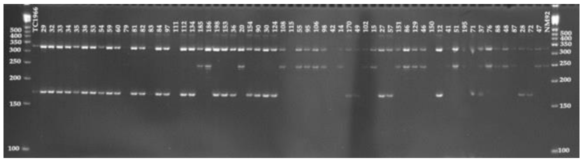

d)

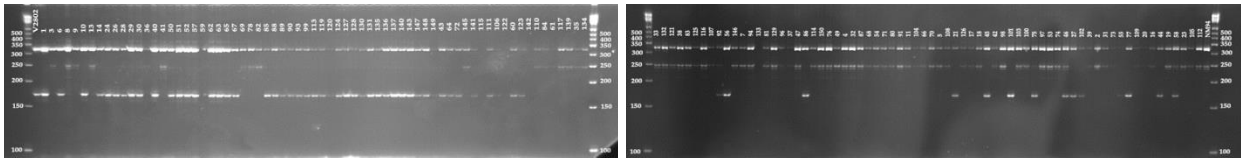

e)

Additional file 1: Genotyping segregating mungbean populations with tetra markers: tetra_6 in population TC1966 x NM92 (a), tetra_7 in V2802 x NM94 (b), tetra_9 in V2802 x NM94 (c), and tetra_12 in TC1966 x NM92 (d) and V2802 x NM94 e).
