# Supplementary material for: PCR-based assays for validation of single nucleotide polymorphism markers in rice and mungbean
Source: Hereditas. 2017 Jan 26;154:3. doi: 10.1186/s41065-016-0024-y (PMC5270362; doi:10.1186/s41065-016-0024-y)
Supplement: Additional file 2: — CEL-I genotyping in segregating mungbean populations TC1966 x NM92 and V2802 x NM94. (DOCX 23250 kb) [file 41065_2016_24_MOESM2_ESM.docx]

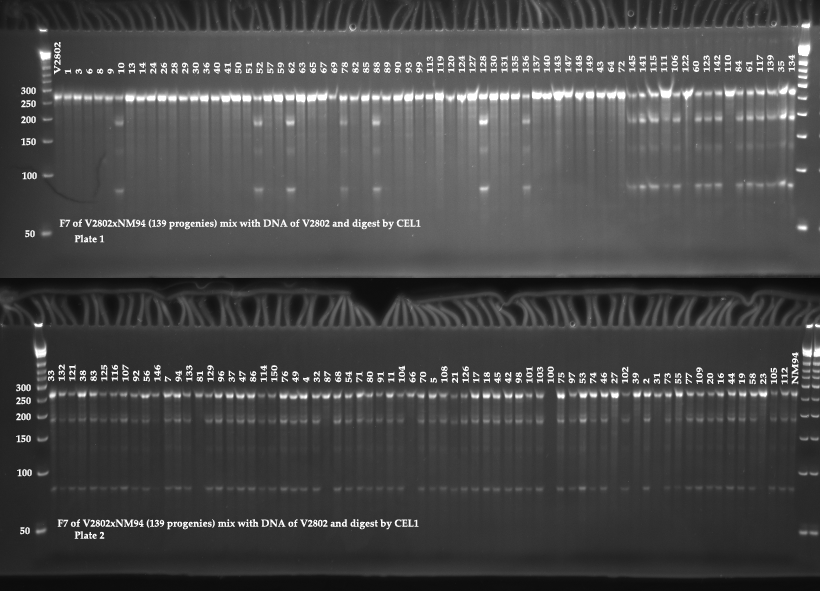

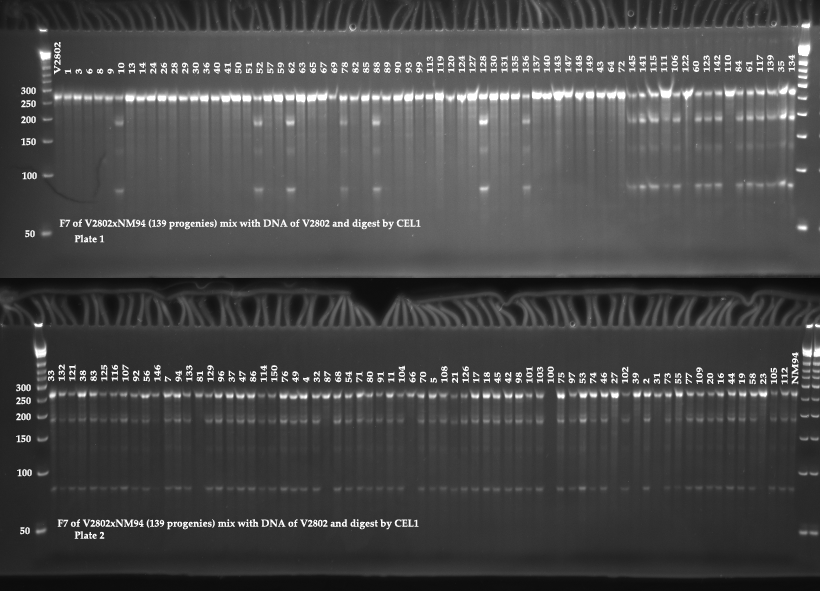


a)


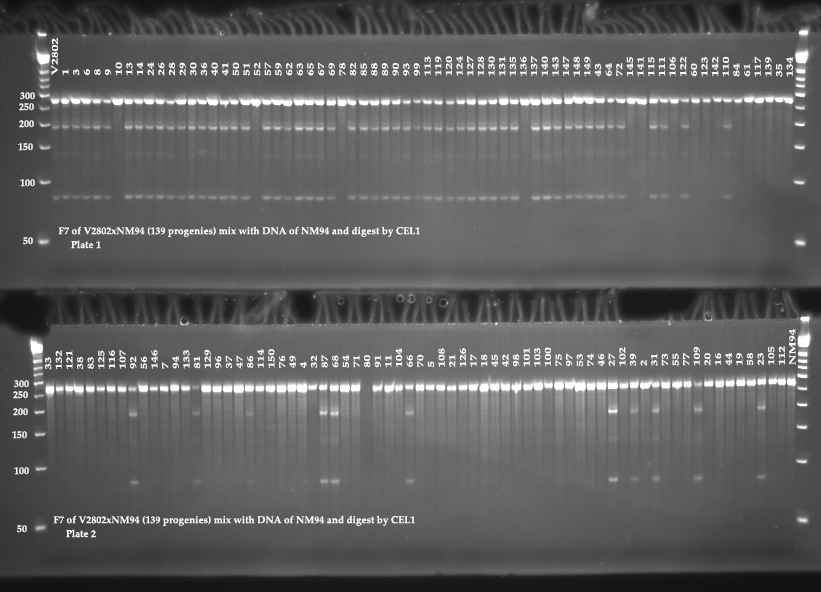

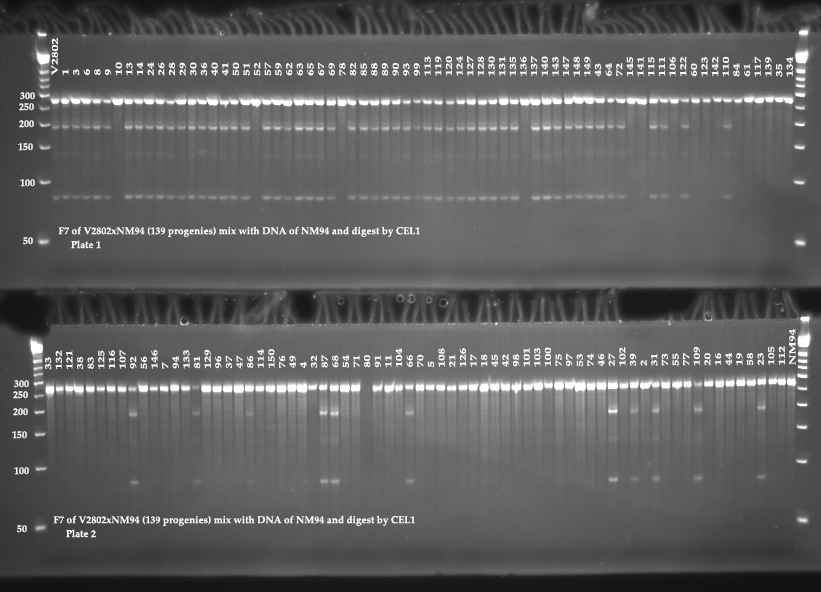


b)


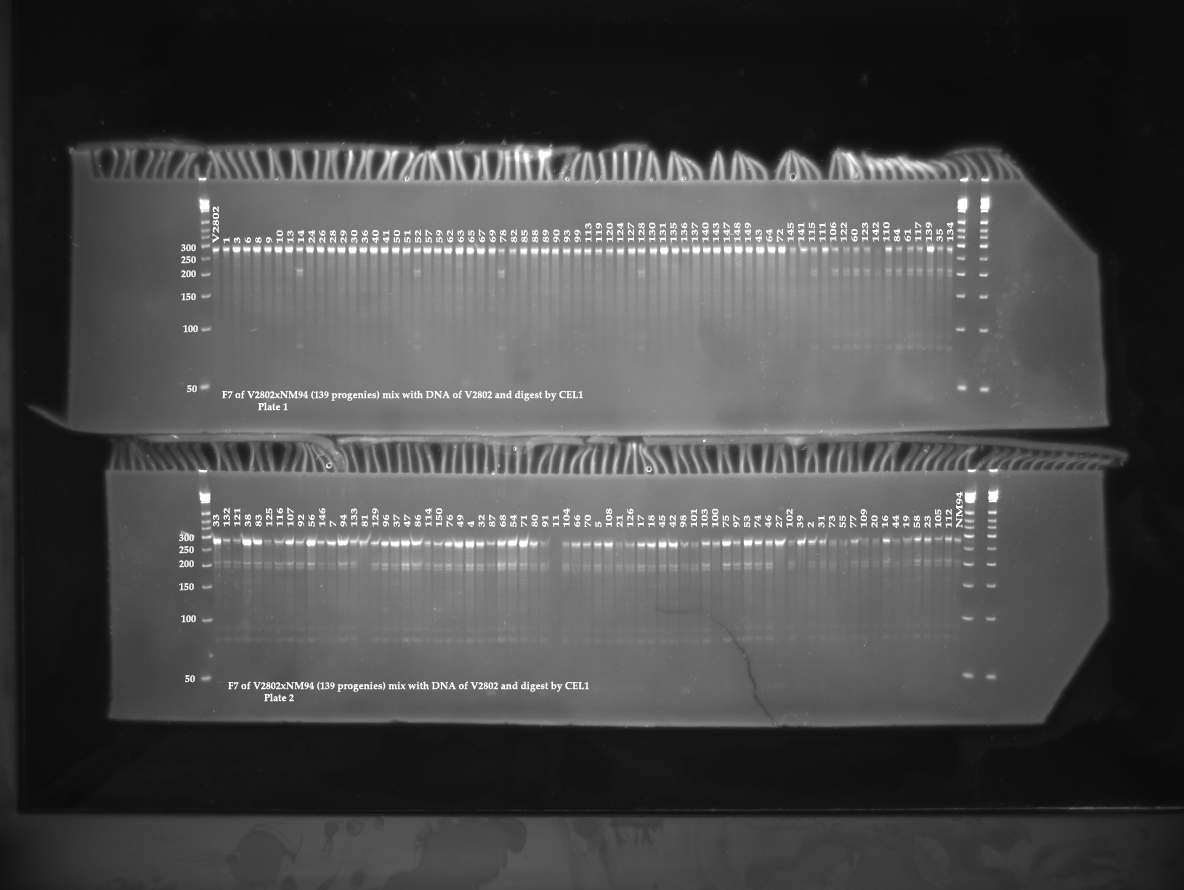

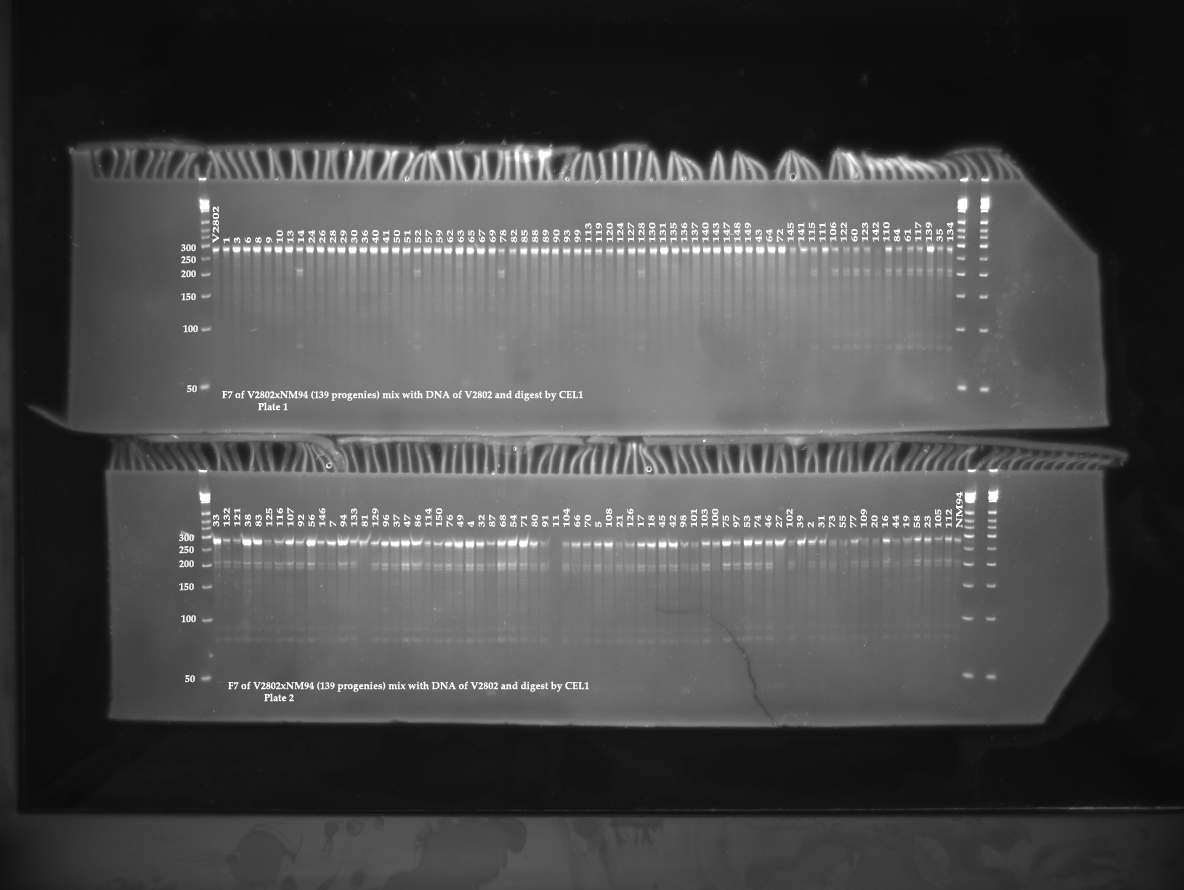


C)


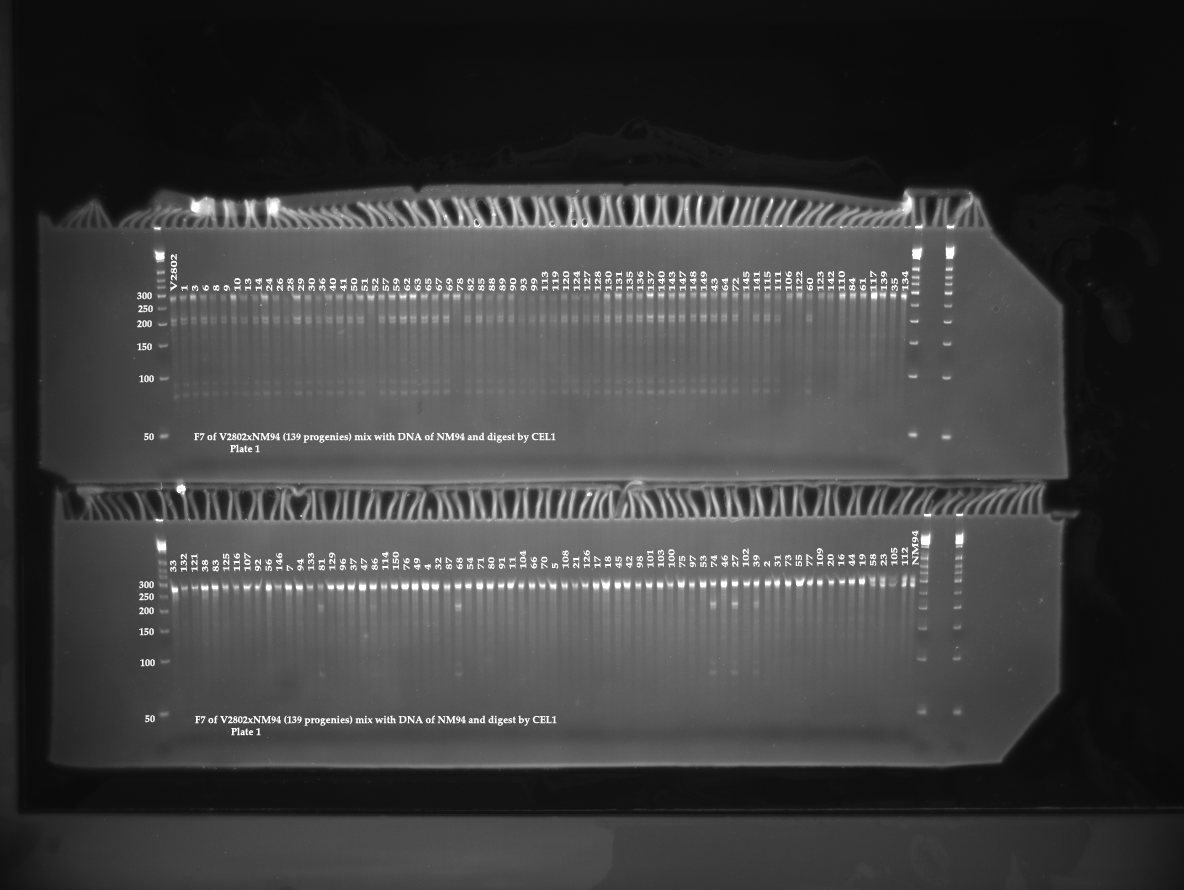

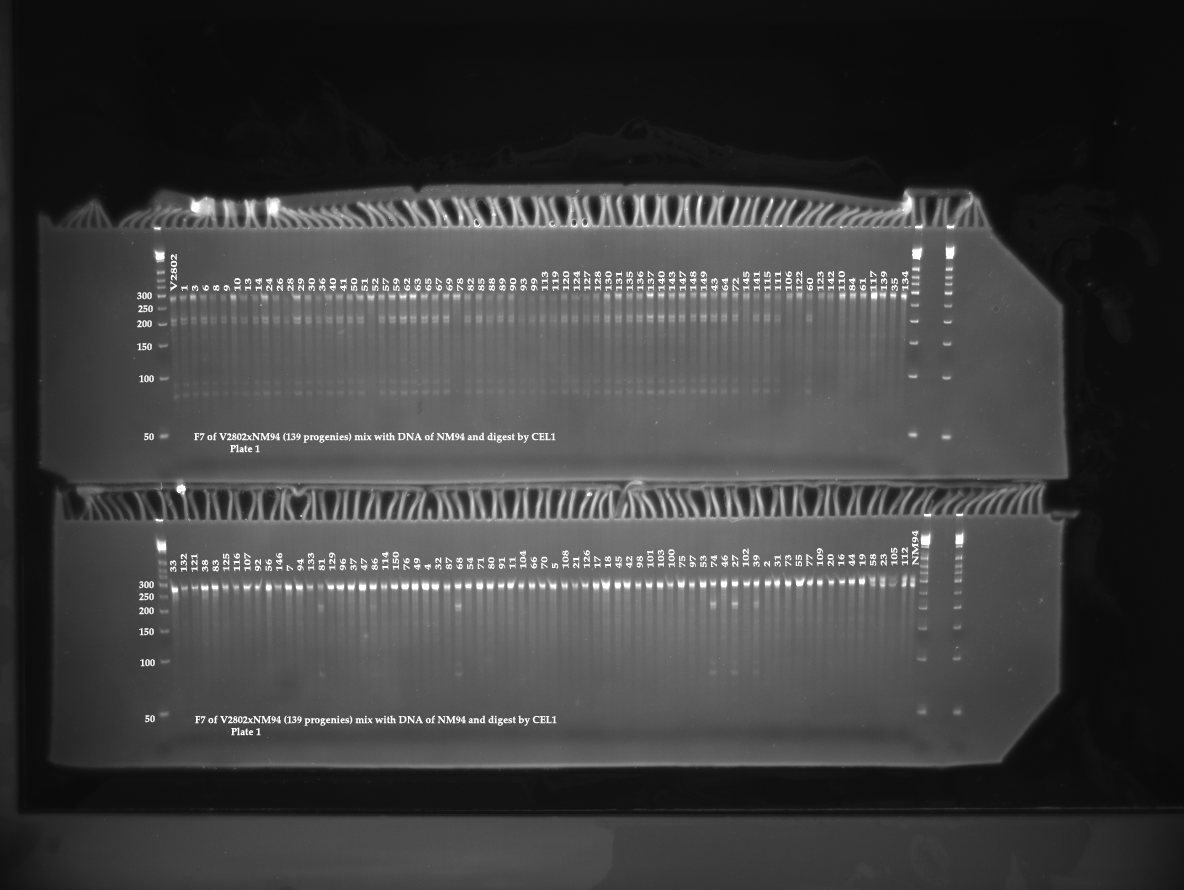


d)


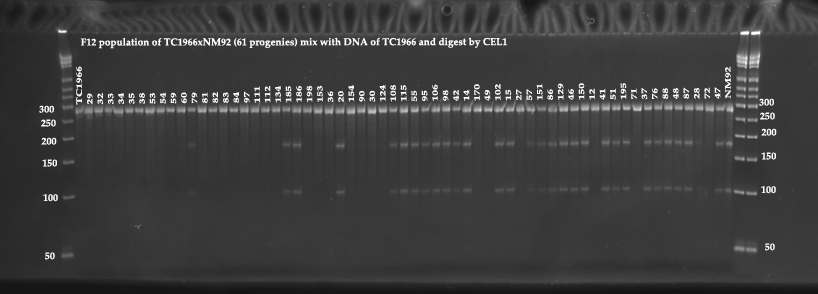


e)


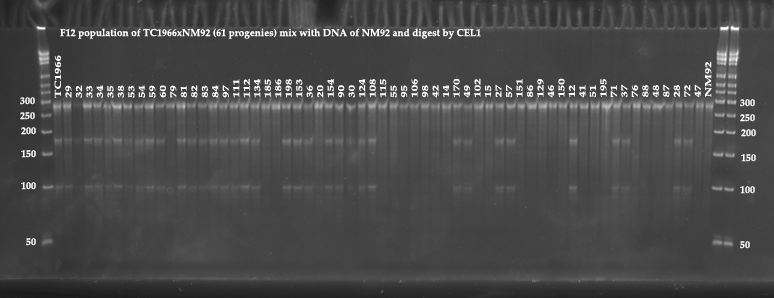


f)


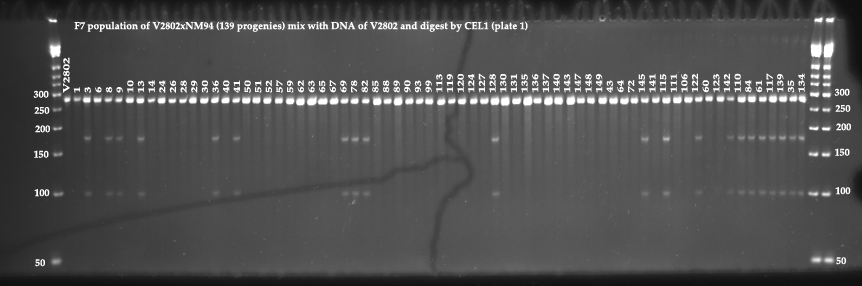

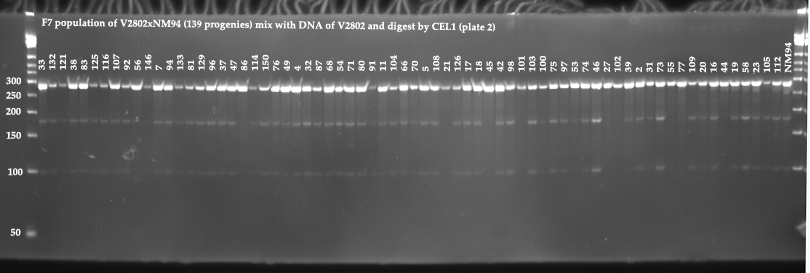


g)


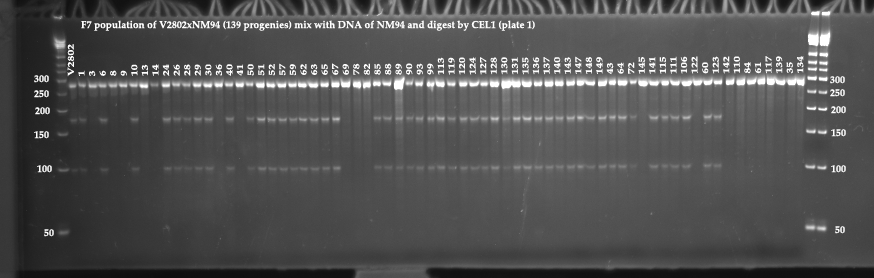

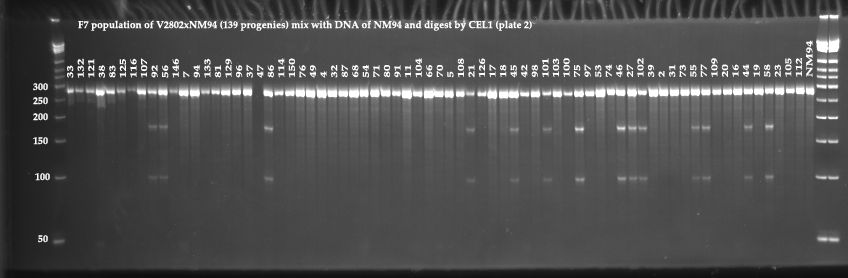


h)

Additional file 3. CEL-I genotyping in segregating mungbean populations TC1966 x NM92 and V2802 x NM94. For each samples, two PCRs and CEL-I digestions were performed, one with the PCR mastermix spiked with parent A DNA, and one with parent B spikes. Cuts present in only one of the two digestions indicate homozygote sites, while heterozygote sites show bands in both digestions.

a) Tetra_7 of V2802 x NM94, spiked with V2802, b) like c but spiked with NM94, c) Tetra_9 of V2802 x NM94, spiked with V2802, d) like c but spiked with NM94, e) tetra 12 of TC1966 x NM92, spiked with DNA of TC1966, f) like e, but spiked with NM92, g) tetra_12 of V2802 x NM94, spiked with V2802, h) like g but spiked with NM94.
